# Resveratrol, a sirtuin 1 activator, increases IL-6 production by peripheral blood mononuclear cells of patients with knee osteoarthritis

**DOI:** 10.1186/1868-7083-5-10

**Published:** 2013-07-11

**Authors:** Daniel Wendling, Wasim Abbas, Marie Godfrin-Valnet, Xavier Guillot, Kashif Aziz Khan, Jean-Pierre Cedoz, Lucile Baud, Clément Prati, Georges Herbein

**Affiliations:** 1 Department of Rheumatology, CHRU de Besançon, Boulevard Fleming, F-25030 Besançon, France; 2 EA 4266, Pathogens & Inflammation Laboratory, SFR FED4234, Université de Franche-Comté, Besançon, France; 3 Department of Virology, CHRU de Besançon, 2 Place Saint-Jacques, F-25030 Besançon, France

**Keywords:** IL-6, Osteoarthritis, PBMCs, Resveratrol, Sirtuin 1

## Abstract

**Background:**

Sirtuin 1 (Sirt1) is a nuclear enzyme from the class III histone deacetylases that modulates gene expression and is involved in bone and cartilage remodeling. The goal of our study was to evaluate Sirt1 activity in peripheral blood mononuclear cells in patients with osteoarthritis in comparison with control patients, and to determine the relationship between Sirt1 activity and production of TNFα, IL-6 and IL-8 by peripheral blood mononuclear cells after *ex vivo* treatment with resveratrol, a Sirt1 activator.

**Results:**

A prospective study was performed to compare the activity of Sirt1 in patients with primary osteoarthritis of the knee (American College of Rheumatology criteria) with its activity in controls. Peripheral blood mononuclear cells were isolated from peripheral blood, and Sirt1 activity evaluated from cytoplasmic and nuclear compartments using a fluorometric assay. Culture supernatant levels of TNFα, IL-6, and IL-8 were quantified before and after resveratrol *ex vivo* treatment. Nineteen patients with symptomatic knee osteoarthritis (age 64 ±9 years) and 18 controls (age 54 ±13 years) were included. No differences were found in cytoplasmic or nuclear Sirt1 activity between patients and controls. After resveratrol treatment, no changes in TNFα or IL-8 levels were found, but a significant dose-dependent increase in IL-6 levels was demonstrated in patients with osteoarthritis, but not controls. Sirt1 activity did not correlate with clinical activity (Lequesne’s index) or inflammation (erythrocyte sedimentation rate, C-reactive protein).

**Conclusion:**

Sirt1 activity (cytoplasmic and nuclear) from peripheral blood mononuclear cells did not differ between patients with osteoarthritis and controls. *Ex vivo* treatment of peripheral blood mononuclear cells with resveratrol was associated with a dose-dependent increase in IL-6 levels only in patients with osteoarthritis.

## Background

Osteoarthritis (OA) is a common chronic age-related disease involving cartilage but also synovial membrane and subchondral bone. It has major functional and socioeconomic impacts. OA is a polygenic disease [1], but epigenetic effects are important mediators of OA biology [2], including DNA methylation and histone modifications. Among histone modifiers, two major classes are recognized; histone acetyl transferases and histone deacetylases (HDAC), with opposite effects [3]. HDACs are divided into four classes. Sirtuins belong to the class III HDACs. Seven types of sirtuins are known, with different molecular targets. Sirtuin 1 (Sirt1) is the most studied of the sirtuins with p53, NF-κB and PGC1α among others as targets. Sirt1 modulates the expression of genes involved in the regulation of various biological processes (cell survival, apoptosis, gluconeogenesis, adipogenesis, lipolysis), and local and systemic inflammation, as well as in bone and cartilage remodeling [4,5]. In the cartilage, Sirt1 modulates chondrocyte apoptosis [6,7] and enhances survival of osteoarthritic chondrocytes [8], and thus may be implicated in the pathogenesis of OA [9]. This ‘protective’ role of Sirt1 is reduced by proinflammatory cytokines such as TNFα, leading to inactivation of Sirt1 in human osteoarthritic chondrocytes [10]. Sirt1 may be activated by several compounds [11], including resveratrol [3]. Activation of Sirt1 by resveratrol has been demonstrated in articular chondrocytes [12], and resveratrol may have a positive effect on cartilage protection and apoptosis inhibition [13].

The hypothesis was that Sirt1 activity is reduced in patients with knee OA compared with healthy controls. This hypothesis was tested exploring an accessible body compartment, namely peripheral blood.

The main objective of the study was to evaluate nuclear and cytoplasmic Sirt1 activity in peripheral blood mononuclear cells (PBMCs) in patients with knee OA in comparison to control patients, via venous blood aspiration. The secondary objectives were to analyze the relationship between Sirt1 activity and production of mediators of inflammation and cytokines (TNFα, IL-6, IL-8) by the cells after *ex vivo* treatment with a sirtuin activator, resveratrol.

## Results

Nineteen patients with symptomatic primary knee OA (age 64 ±9 years; mean Lequesne’s index: 8.4; grade II or III of the Kellgren-Lawrence classification) and 18 controls (age 54 ±13 years) were included. No differences were found between patients and controls in cytoplasmic (*P* = 0.8) or nuclear (*P* = 0.5) Sirt1 activity (Table 1). There was no correlation between Sirt1 activity (nuclear and cytoplasmic) and biologic inflammation (erythrocyte sedimentation rate, C-reactive protein). Sirt1 activity did not correlate with clinical activity assessed by Lequesne’s index (*P* = 0.8).

**Table 1:** Values of biological parameters in patients with osteoarthritis and control participants

**Mean ±SD**	**Sirt1 nuclear activity**	**Sirt1 cytoplasmic activity**	**C-reactive protein (mg/l)**	**Erythrocyte sedimentation rate (mm/h)**	**TNFα (pg/ml)**	**IL-6 (pg/ml)**	**IL-8 (pg/ml)**
**Control**	382,825 ± 191,023	352,907 ± 195,317	2.6 ± 1.7	3.0 ± 14.5	29.0 ± 27.2	8.6 ± 5.0	1,083.3 ± 771.6
**Patients**	348,713 ± 216,232	316,314 ± 217,313	14.6 ± 11.0	4.5 ± 2.7	23.7 ± 21.4	5.1 ± 3.6	1,148.7 ± 2,173
***P***	0.5	0.8	0.0002	0.01	0.5	0.03	0.3

**Figure 1:**
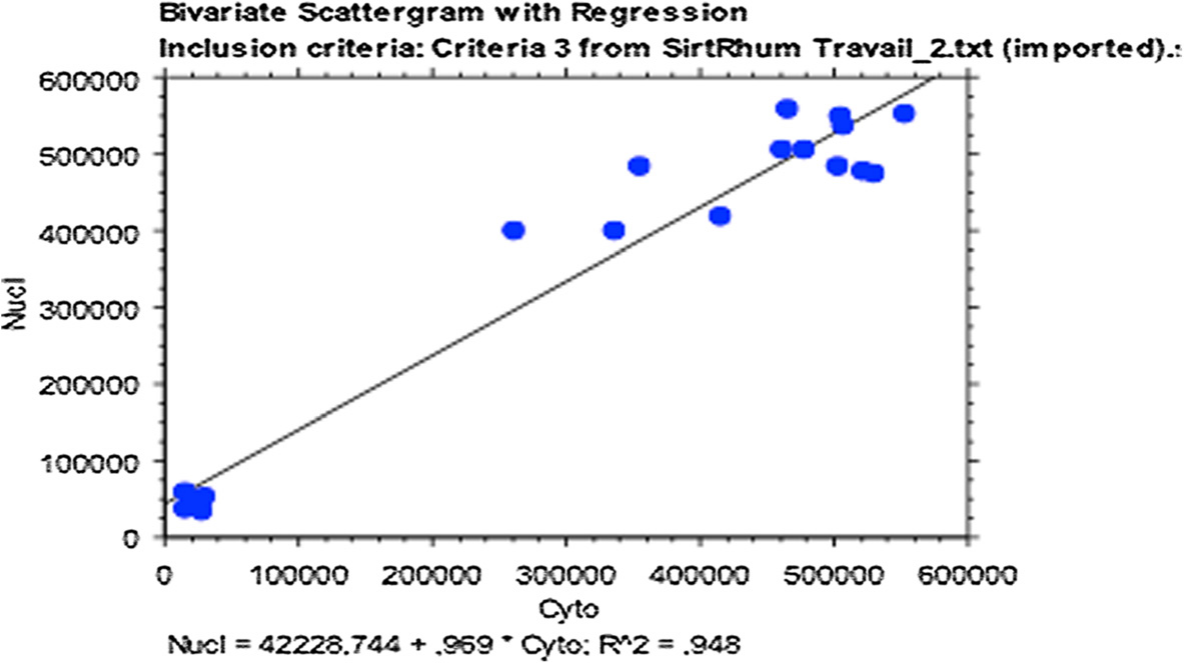
**Correlation between nuclear and cytoplasmic Sirt1 activity in patients with osteoarthritis (R**^**2 **^**= 0.948).**

**Figure 2:**
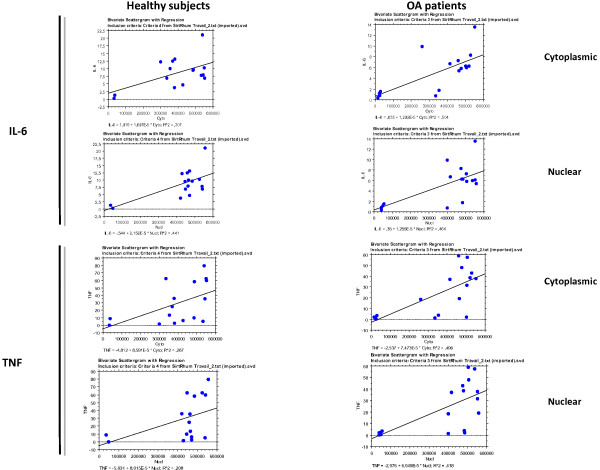
Correlation between Sirt1 activity (nuclear and cytoplasmic) and baseline IL-6 and TNFα in controls and patients with osteoarthritis.

**Figure 3:**
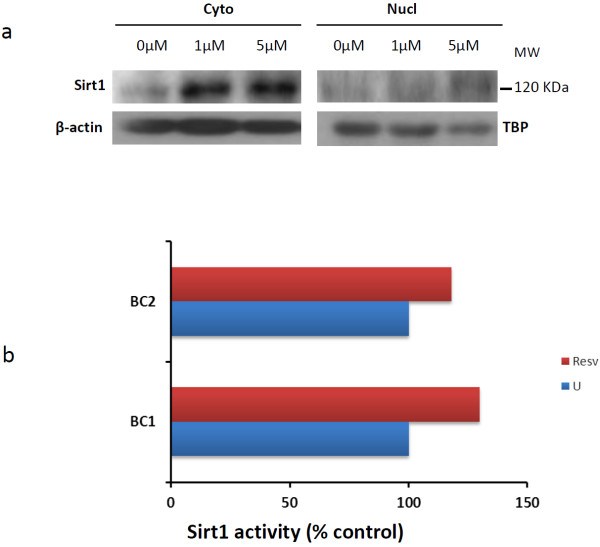
**Effect of resveratrol on sirtuin 1 activity and expression in healthy controls. (a)** Enhanced Sirt1 protein expression and **(b)** Sirt1 activity in peripheral blood mononuclear cells of healthy controls treated with resveratrol for 48 hours (at 1 μM and 5 μM for western blot and at 5 μM for cytoplasmic Sirt1 activity). BC, buffy coat; TBP, TATA-binding protein.

**Figure 4:**
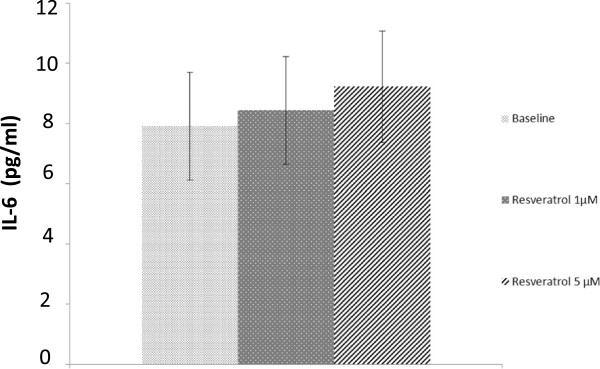
**Concentration of IL-6 in supernatants of peripheral blood mononuclear cells isolated from patients with osteoarthritis and treated *ex vivo *by resveratrol at 1 μM and 5 μM (*P *= 0.02).**

We found a correlation between cytoplasmic and nuclear Sirt1 activity in both populations (patients with OA and controls; R^2^ = 0.948) (Figure 1). Sirt1 activity (nuclear and cytoplasmic) was correlated to baseline IL-6 (*P* = 0.002) and baseline TNFα (*P* = 0.004) but not with IL-8 (*P* = 0.74) (Figure 2 and data not shown). In agreement with the up-regulation of both Sirt1 protein expression and Sirt1 activity by resveratrol in several cell types [14,15], we observed a similar increase of both Sirt1 protein expression and activity in PBMCs isolated from healthy donors (buffy coats) and treated with resveratrol (Figure 3). After resveratrol treatment, no changes in TNFα or IL-8 levels were found, but a significant dose-dependent increase in IL-6 levels in the supernatants of PBMCs cultured for 48 h was demonstrated in patients with OA (*P* = 0.02; Figure 4), but not in controls.

## Discussion

The purpose of this study was to evaluate Sirt1 activity in cells from the peripheral blood compartment, which is more easily accessible than cartilage tissue.

This study shows that Sirt1 evaluation on PBMCs drawn from peripheral blood is feasible, allowing an accessible cell subset for investigation. Moreover, this study demonstrates that Sirt1 activity is present, not only in the nuclear compartment but also in the cytoplasmic one, and both are correlated. Although the literature has previously shown mainly the nuclear activity of Sirt1 [3], at least two different mechanisms could account for its biological action. Sirt1 preferentially deacetylates lysine 9 of histone H3 and lysine 16 of histone H4 [16]. Additionally, Sirt1 interacts directly with the p65 subunit of NF-κB, leading to deacetylation at lysine 310, culminating in decreased NF-κB-associated transcription [17]. Sirt1 deacetylates lysine 310 of RelA/p65 without affecting the acetylation status of other lysine residues [17]. Following resveratrol treatment, the localization of both Sirt1 and RelA/p65 proteins on the gene promoter suggests that Sirt1 may actively repress gene expression by deacetylating RelA/p65 directly on chromatin [16]. In the case of NF-κB, the heterodimer composed of RelA/p65 and p50 proteins interacts also with HDAC1, 2 and 3 enzymes, making the regulation of NF-κB-dependent genes even more complex [18]. Recently, the cathepsin B-mediated cleavage of Sirt1 by TNFα has been reported [10]. Interestingly, other proinflammatory cytokines participating in arthritis, including IL-6, have been reported to activate cathepsin B and therefore could also trigger the cleavage of Sirt1 [19].

It has been demonstrated that Sirt1 regulates apoptosis- and cartilage-specific gene expression in human chondrocytes and mouse models [20]. Mice without Sirt1 activity are characterized by reduced levels of type II collagen, aggrecan, glycosaminoglycan, and elevated levels of matrix metalloproteinases 8, 9 and 13 in the cartilage, and elevated chondrocyte apoptosis. Normal cartilage homeostasis requires enzymatically active Sirt1 protein. Impaired Sirt1 activity may favor the development of OA.

An impairment of Sirt1 activity was hypothesized in patients with OA patients, however no difference was found between patients with OA and controls in regard to nuclear and cytoplasmic Sirt1 activity. The significantly younger age of the controls does not explain this result, since Sirt1 activity is supposed to decrease with age. Nevertheless, several other factors including diabetes and obesity have been reported to modulate Sirt1 activity [21]. No correlations with erythrocyte sedimentation rate or C-reactive protein were found in our study, but we found a correlation between Sirt1 and TNFα and IL-6. Moreover, after resveratrol treatment, a dose-dependent increase of IL-6 in the supernatants of PBMCs was found in patients with OA only, without a change in TNFα levels. Resveratrol enhances the deacetylase enzymatic activity of Sirt1 [22]. We observed that resveratrol at 1 μM and 5 μM increased the expression of Sirt1 protein in PBMCs isolated from buffy coats from healthy donors using western blotting (Figure 3a). Additionally, the activity of Sirt1 was increased in PBMCs treated with 5 μM resveratrol (Figure 3b). In agreement with our data, other studies report the up-regulation of Sirt1 expression and Sirt1 activity in primary human endothelial cells or human HepG2 hepatocytes treated with 10 μM resveratrol [14,22]. Resveratrol at 50 nM inhibits TNFα-induced inflammation measured by matrix metalloproteinase-9 expression in 3T3/NIH cells [15]. Altogether, our data and those of others indicate a biological effect of resveratrol at concentrations used in our study (1 to 5 μM).

Recent reports indicate that Sirt1 can stimulate proinflammatory cytokine production [23,24]. In agreement with increased IL-6 production in response to resveratrol treatment in PBMCs, HDAC inhibitors inhibit IL-6 release by bone marrow-derived macrophages exposed to microbial products such as lipopolysaccharides (LPS) and heat-killed *Escherichia coli* and *Staphylococcus aureus*[25,26]. Sirtuin inhibition decreases the production of TNFα, IL-6 and regulated upon activation normal T cell expressed and secreted (RANTES) in LPS-stimulated macrophages [23], suggesting that sirtuin activation may favor proinflammatory cytokine production by activated macrophages. In agreement with this hypothesis, inhibition of Sirt1 enzymatic activity reduces LPS-induced levels of TNFα in monocytes of patients with rheumatoid arthritis [24]. By contrast, other studies indicate that resveratrol inhibits TNFα-induced inflammation via Sirt1 [15], and suppresses expression of TNFα, IL-6 and IL-8 [27]. Sirt1 deacetylates the p65 subunit of NF-κB at lysine 310, attenuating NF-κB transcriptional activation, and thereby could decrease proinflammatory cytokine production. Altogether, Sirt1 activity and subsequent proinflammatory cytokine production may depend on the cell type involved (monocytes or macrophages, or PBMCs in peripheral blood compartment versus cells of the cartilage tissue) and on the activation state of the cell type studied (unstimulated cells versus LPS-activated cells).

Some findings of our study deserve to be highlighted. We were able to measure Sirt1 activity in the cytoplasmic compartment, and found a correlation with nuclear Sirt-1 activity. This demonstrates that Sirt1 activity may be assessed in PBMCs, allowing more easy access than cartilage tissue in humans, and thus could favor repetitive and sequential evaluations of treatments in the future. Additionally, because Sirt1 is involved in cartilage biology, it could be a major target for future therapies [28].

## Conclusion

Sirt1 activity (cytoplasmic and nuclear) from PBMCs was not different between patients with OA and controls. Nevertheless, *ex vivo* treatment of PBMCs with resveratrol, a Sirt1 activator, was associated with increased IL-6 levels in a dose-dependent manner only in the patients with OA, suggesting that IL-6 expression could be specifically regulated via Sirt1 in OA. Importantly, Sirt1 activity may be assessed in PBMCs, in the nuclear as well as in the cytoplasmic compartment.

## Methods

A prospective and comparative monocentric study was performed to compare the activity of Sirt1 in patients with OA and controls, after written informed consent. The protocol was approved by the local ethics committee (Comité de Protection des Personnes-Est 2). Inclusion criteria were: patients aged 18 to 80 years, with symptomatic primary knee OA defined according to the American College of Rheumatology criteria, with radiological grading (Kellgren-Lawrence classification over I). Exclusion criteria were: immunosuppressive drugs, diabetes or neurodegenerative disease. The control group consisted of healthy volunteers. Symptoms were quantified with Lequesne’s algofunctional index for knee OA.

PBMCs were isolated by Ficoll gradient centrifugation. Blood from a patient or a healthy donor was diluted with equal amounts of PBS, overlaid on Ficoll medium (Eurobio, Les Ulis, France) and centrifuged at 900 X g for 30 min at 25°C. The PBMC band was removed and washed twice with PBS. Cell count was determined by Malassez cytometry (Poly Labo, Strasbourg, France), and cells were resuspended in serum-free Roswell Park Memorial Institute medium 1640.

Isolation of nuclear and cytoplasmic extracts was performed as follows. After isolation, PBMCs were harvested and washed with wash buffer (10 mM HEPES (pH 7.6), 10 mM KCl, 2 mM MgCl_2_, 1 mM EDTA). Cell pellets were then incubated on ice with cytoplasmic isolation buffer (10 mM HEPES (pH 7.6), 10 mM KCl, 2 mM MgCl_2_, 1 mM EDTA, 0.02% Nonidet P-40). Cytoplasmic extracts were collected by centrifugation, and the nuclear pellets were washed twice in wash buffer, spun, and incubated for 15 min on ice with nuclear isolation buffer (20 mM HEPES (pH 7.6), 420 mM NaCl, 1.5 mM MgCl_2_, 0.2 mM EDTA, 25% glycerol). Supernatants containing nuclear extracts were collected by centrifugation and stored at -80°C. Protease inhibitors (1 mM DTT, 1 mM PMSF, 1 μg/ml aprotinin, 1 μg/ml leupeptin, 1 μg/ml pepstatin) were added to all solutions. Protein concentration in nuclear and cytoplasmic extracts was determined by the Bradford method using a Bio-Photometer (Eppendorf, Hamburg, Germany). The purity of cytoplasmic and nuclear extracts was further confirmed by the quantification of the expression of β-actin, a cytoplasmic marker, and TATA-binding protein, a nuclear marker, using western-blotting (data not shown).

For western blot analysis, 10 μg of cellular extracts were resolved on 10% SDS-PAGE using a Mini-PROTEAN 3 Cell (Bio-Rad Laboratories, Hercules, CA, USA). The proteins were electrotransferred onto a polyvinylidene difluoridemembrane (Amersham Biosciences, Saclay, France) using Mini Trans-Blot Electrophoretic Transfer Cell (Bio-Rad Laboratories). The membranes were probed with primary antibodies followed by horseradish peroxidase-conjugated secondary immunoglobulin raised against the appropriate species; bands were detected using the ECL Plus kit (Amersham Biosciences). The primary antibodies used for western blot are as follows: rabbit anti-Sirt1 antibody (Cell Signaling Technology, Beverly, MA, USA); and mouse anti-β-actin antibody and mouse anti-TATA-binding protein antibody (Sigma-Aldrich, St. Louis, MO, USA). Horseradish peroxidase-conjugated secondary antibodies goat anti-rabbit (Santa Cruz Biotechnology Inc, Santa Cruz, CA, USA) and rabbit anti-mouse (DakoCytomation, Trappes, France) were used.

Sirt1 activity was evaluated from cytoplasmic and nuclear compartments using a fluorometric assay (SIRT1 fluorimetric kit, BML-AK-555; Enzo Life Sciences, Villeurbanne, France) at the 15-min point. PBMC culture supernatant levels of TNFα, IL-6 and IL-8 were quantified at 48 hours without and after resveratrol (1 μM and 5 μM) *ex vivo* treatment, with commercial kits (Quantikine Kits, R&D Systems, Minneapolis, MN, USA), according to the manufacturer’s recommendations. Statistical analysis was carried out using Wilcoxon and t tests with significance set at *P <*0.05.

## Abbreviations

HDACs: Histone deacetylases; LPS: Lipopolysaccharide; NF-κB: Nuclear factor-kappa B; OA: Osteoarthritis; PBMCs: Peripheral blood mononuclear cells; PBS: Phosphate-buffered saline; Sirt1: Sirtuin 1; TNFα: Tumor necrosis factor-alpha.

## Competing interests

GH is a member of the editorial board of Clinical Epigenetics. All other authors declare that they have no competing interests.

## Authors’ contributions

WA and KAK processed the blood samples, and prepared cytoplasmic and nuclear extracts; LB performed the sirtuin assay; MG-V, XG, JPC and CP enrolled the patients; DW and GH designed the experiments and wrote the manuscript. All authors read and approved this manuscript.
